# Supplementary material for: Identification of photoperiod-induced specific miRNAs in the adrenal glands of Sunite sheep (Ovis aries)
Source: Front Vet Sci. 2022 Jul 22;9:888207. doi: 10.3389/fvets.2022.888207 (PMC9354845; doi:10.3389/fvets.2022.888207)
Supplement: Supplementary file 1 [file Data_Sheet_1.ZIP › Supplementary file/miRDeep2 file.zip!/miRDeep2 file/Know_Structure/SPLP42A2/oar-mir-106a.pdf]

miRBase precursor : oar-mir-106a
Total read count : 65
oar-miR-106a read count : 65
remaining reads : 0

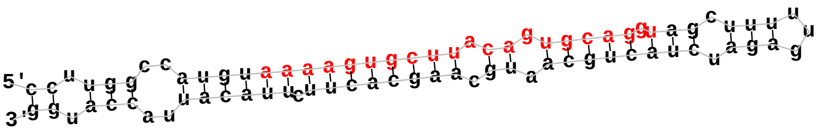

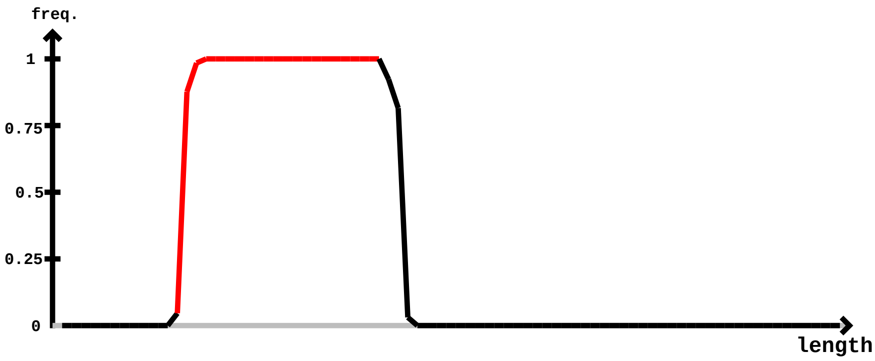


```
               oar-miR-106a
5'- ccuuggccauguaaaagugcuuacagugcagguagcuuuuugagaucuacugcaaugcaagcacuucuuacauuaccaugg   -3'    exp
    ((.(((..((((((((((((((.((.(((((.(((.(((...))).)))))))).)).)))))))).))))))..))).))    reads      mm         sample
    ...........uaaaagugcuuacagugcaggua...............................................    1          0          seq
    ...........uaaaagugcuuacagugcagguag..............................................    2          0          seq
    ............aaaagugcuuacagugcaggu................................................    4          0          seq
    ............aaaagugcuuacagugcaggua...............................................    5          0          seq
    ............aaaagugcuuacagugcagguag..............................................    43         0          seq
    ............aaaagugcuuacagugcagguagc.............................................    1          0          seq
    ............aaaagugcuuacagugcagguagG.............................................    1          1          seq
    .............aaagugcuuacagugcaggu................................................    1          0          seq
    .............aaagugcuuacagugcaggua...............................................    1          0          seq
    .............aaagugcuuacagugcagguag..............................................    5          0          seq
    ..............aagugcuuacagugcagguag..............................................    1          0          seq
```
